# Charting Professional Waters: Diary of a Young Psycho-Oncologist

**DOI:** 10.1200/GO.23.00357

**Published:** 2023-11-16

**Authors:** Bincy Mathew

**Affiliations:** ^1^ Manipal Comprehensive Cancer Care, Bangalore, India

## Abstract

A young psycho-oncologist faces a moral dilemma, testing professional ethics against personal morals.

It was a regular day toward the end of the year 2016 at the Tertiary Cancer Centre in Chennai, India, where I was completing my MPhil studies as a psycho-oncologist (a psycho-oncologist supports the mental well-being of patients with cancer during diagnosis, treatment, transitioning to and throughout survivorship, and approaching the end-of-life). We were on our regular rounds at the patient wards, and suddenly my gaze fell on Saraswati (name changed), a 5-year-old girl, and her mother Arumai (name changed), a woman in her early thirties. This mother and child duo was different from any other patients I had ever encountered, and for some reason, a wave of childhood memories with my own mother came flooding back, creating an immediate bond between us.

In the midst of the bustling and frenzied oncology out-patient department, their warm smiles and serene composure struck me as almost surreal with their peaceful presence as a welcome respite. The gravity of the situation did not hit me until my colleague whispered that Saraswati had been diagnosed with AML. There was no sign of worry on Arumai's face—she seemed to know exactly what her daughter was up against. In the following days, I found myself drawn deeper into Saraswati's world as I listened intently to her mother's stories. They were from a humble background in a rural village in Tamil Nadu, India, and Saraswati had an infectiously lively and carefree spirit. Her mother described her as a bundle of joy, and she loved exploring everything around her at the hospital, making friends easily. Her father, the sole breadwinner of the family, was a daily wage worker struggling to make ends meet. The initial treatment had already placed them in a dire financial situation, and the prospect of continued expenses was daunting. Her father and the paternal side of the family were adamant that they should not spend more money on her treatment, but Arumai refused to give up hope.

Despite their low socioeconomic status, she was determined to do everything she could to save her daughter's life. As our connection grew deeper, Arumai increasingly confided in me more and more. Almost every conversation ended with her desperate plea for reassurance that her beloved Saraswati would recover. I knew it was her way of seeking strength, and all I could offer was assurance of the hospital's and doctor's expertise and hope for the best. In spite of my lack of concrete answers, she continued to trust me with her deepest fears and hopes.

Over time, she took me through the whole story from the very beginning. It all started with a recurrent fever that disrupted Saraswati's ability to attend school and eventually led to her being diagnosed with cancer. In a male-dominated society where men traditionally handle travel and financial matters, women from rural areas like Arumai face challenges when dealing with city-related tasks. This includes finding accommodation, late-hour travel, and navigating busy hospitals, sometimes leaving their children unattended. She recounted many ordeals about multiple trips to a regional cancer center in Chennai, Tamil Nadu, where Saraswati was placed under the care of a pediatric oncologist. Despite her limited education, Arumai was well-informed about the disease and treatment protocols, as if being so would give her daughter the best chance of surviving. As the treatment progressed and the side effects piled up, Saraswati spoke less than before, but her bright smile never wavered. Saraswati's hair loss was particularly difficult for her to come to terms with. Once adorned with beautiful light brown hair neatly braided to both sides, she would innocently ask me if her hair would ever grow back. In her distress, she would delicately touch my own hair, conveying her emotions without words.

After induction, Saraswati showed a poor response to the treatment. The protocol was modified, but her disease remained refractory. A stem-cell transplant was the only viable option, and after many months of waiting, a suitable donor was finally found. Although the treatment they received was heavily subsidized, patients still had some out-of-pocket expenses to bear. Fortunately, the delay in their treatment allowed Arumai to secure financial assistance from a non-governmental organization, which came as a significant relief to her. The news about the match brought everyone a sudden burst of hope and optimism. The pediatric oncologist explained the entire process of bone marrow transplant to Saraswati and her family, and I conducted pretransplant counseling sessions to help prepare them psychologically and emotionally for the rigors ahead.

Post-transplant, I continued to check in with them weekly to monitor their progress and offer support. It was a relief to see Saraswati coping well with the treatment and the hope it brought for a better future. Despite this, I could not ignore the deep sadness that hung over Arumai. I learned that her husband had abandoned his own family, cutting off all communication and leaving them to fend for themselves. Consequently, they had to relocate to cheaper lodging, securing a place for just 100 Indian rupees ($1.2 US dollars) per day.

After the successful transplant, their decision to stay in the city for scheduled follow-up visits was influenced by Saraswati's concern about burdening others. Because of the societal stigma linked to cancer and the financial strain it placed on her family, Saraswati already felt like a burden within her household. She believed it would be wiser to distance herself rather than exacerbate the perception that she was the cause of their difficulties. After 6 months of battling post-transplant complications, Saraswati's condition took a turn for the worse, and she was readmitted to the hospital. One day, during a mid-day meal with colleagues, I unexpectedly received an urgent call to meet with the physician. I rushed to the ward, only to be met with devastating news: Little Saraswati had died. The physician requested my presence to support her mother, and at that moment, I realized that I, too, was experiencing this heart-wrenching moment. I gathered all my strength and walked into the room. I could feel the weight of the loss bearing down on me. The moment I met Arumai, we locked eyes, but she remained silent and motionless, not even blinking. Her eyes were swollen and red. It felt like a flood of emotions that would pour out anytime, but her lips were tightly pressed together, forming a thin, white line that belied the agony she was feeling. Her body still trembled with the aftershocks of grief as she struggled to catch her breath. All that remained was the overwhelming sense of loss and the crushing weight of sorrow. It was clear that she was not just grieving for her child but for the future that had been stolen from them both. In that moment, I found no words of wisdom and could not fathom the depths of her pain because deep inside, I was also mourning the loss of my little friend.

As the evening dragged on, the nurse arrived to discuss the cremation procedures. Without hesitation, my colleague and I made the decision to stay with her throughout, doing our best to provide whatever solace we could as she struggled to come to terms with the reality of her daughter's death. The following day, Saraswati's uncle arrived from their hometown, offering a much-needed source of support during this difficult time. With their finances already stretched thin, the uncle selflessly offered to take care of the cremation. His small act of kindness provided much-needed relief, helping to ease the burden of Arumai's grief-stricken heart. We waited outside before the arrival of Saraswati's body. When she finally arrived on a stretcher and moved into the ambulance, Arumai stepped forward and requested permission to hold her daughter's body in her lap during the short journey. When we reached the location of the cremation center, we learned that women were not allowed inside during the pooja (a religious ritual, ceremony, or worship practice in the Hindu culture).

Arumai was forced to part ways with her daughter as she was taken inside. The poojari (Hindu priest) conducted the necessary offerings and rituals as we waited outside. Despite the weight of her loss, not a sound escaped her lips still. Not a single tear fell from her eyes. The grief that consumed her was palpable, and our hearts ached in unison as we wondered how she was holding herself together. As the ritual concluded, the poojari approached them carrying kalash—the clay pot, filled with Saraswati's bones and ashes. As soon as Arumai took the pot in her hand, her body seemed to betray her and her muscles were unable to bear the weight of her sorrow as she crumpled to the ground. The intensity of her emotions were simply too much to bear as she shook with uncontrollable sobs and gasped for air. It was a heart-wrenching sight that left me feeling utterly helpless. My heart pounded fiercely in my chest as I watched her agony unfold, feeling as though I too were being shattered by the sheer force of her pain.

In just a matter of hours, a mother had gone from holding her daughter to holding a pot filled with her ashes. She had done everything possible to help her daughter, yet she was left with nothing but questions for God. She had faced the heart-breaking decision to uproot her life and move to a different city for her daughter's treatment, only to end up with this devastating outcome. Not knowing what else to do or say, I simply sat and wrapped my arms tightly around her. It was my only way of offering some comfort in that dark moment.

As a psychologist specializing in end-of-life care, I have faced my fair share of difficult situations, but none have left me feeling as overwhelmed and helpless as this one. Every moment since has felt like a cruel reminder of life's unfairness and unpredictability. I found myself questioning my own abilities and struggling to maintain my personal and professional boundaries. I felt a desperate need to help this individual, whose situation was dire, and yet the more I tried, the more helpless I felt.

Days turned into weeks, and I found myself lost in a storm of conflicting emotions, and it took all of my strength to keep from drowning in a sea of despair. I knew these emotions were taking a toll and I finally reached out for professional help. Looking back at the whole ordeal, I can now see that this experience has changed me in ways that I never could have imagined. As a human being, I still take comfort in knowing that I was there for that mother. All I could do was hold on and try to weather the storm, hoping that somehow, some way, I could make a difference in the life of the person who so desperately needed my help. At the same time, I am grateful for going through such an intense situation early on in my professional career. I have gained invaluable insights into how intense experiences can profoundly affect us in this profession. I have questioned the need for rigid boundaries in our daily work as cancer care providers as each of us possess unique abilities to help those dealing with trauma through our visceral and emotional responses. I have come to understand that while we should maintain strong principles and guidelines, we should also be flexible in serving those in need. It is acceptable to occasionally extend beyond our usual boundaries, but it is crucial to acknowledge the potential repercussion and, more importantly, be willing to seek support.

Seeking supervision from senior therapists was incredibly helpful in my healing process. This case was a true testimonial of how they help cultivate resilience, improve mental well-being, and navigate the emotional complexities of this profession. Neglecting this aspect will eventually pull us into a spiral of self-doubt and negativity, leading us to feel like impostors. As a psycho-oncologist, seeking personal and professional help when needed is an important part of self-care and should not get lost in the chaos of a busy work life or buried under preconceived notions of our own resiliency. Seeking help is not a sign of weakness but a step toward a healthier and more fulfilling professional and personal life. One that benefits not just us, but also those we serve. I am truly grateful to Arumai and Saraswati for their role in my early career as they placed me in a situation that awakened my awareness to the realities of my profession. While I may have encountered many other special families and challenging cases, this one, particularly, resonated deeply with me and offered a profound insight into the true beauty and heartbreak of this field.

